# An electrocardiographic score to predict pulmonary hypertension in children with atrial septal defect

**DOI:** 10.1186/s12887-023-04102-1

**Published:** 2023-06-10

**Authors:** Indah K Murni, Taichi Kato, Muhammad Taufik Wirawan, Nadya Arafuri, Kristia Hermawan, Anggoro Budi Hartopo, Dyah Wulan Anggrahini, Sasmito Nugroho, Noormanto Noormanto, Noriaki Emoto, Lucia Kris Dinarti

**Affiliations:** 1 Department of Child Health, Faculty of Medicine, Public Health and Nursing, Universitas Gadjah Mada, Dr. Sardjito Hospital, Yogyakarta, Indonesia; 2 Centre for Child Health-Pediatric Research Office (CCH-PRO), Faculty of Medicine, Public Health and Nursing, Universitas Gadjah Mada, Yogyakarta, Indonesia; 3 Department of Pediatrics/Developmental Pediatrics, Nagoya University Graduate School of Medicine, Nagoya, Japan; 4 Department of Cardiology and Vascular Medicine, Faculty of Medicine, Public Health and Nursing, Universitas Gadjah Mada, Dr. Sardjito Hospital, Yogyakarta, Indonesia; 5 Division of Cardiovascular Medicine, Department of Internal Medicine, Kobe University Graduate School of Medicine, Kobe, Japan; 6 Laboratory of Clinical Pharmaceutical Science, Kobe Pharmaceutical University, Kobe, Japan

**Keywords:** Electrocardiography, Atrial septal defect, Pulmonary hypertension, Indonesia, Congenital heart disease, Predictive score

## Abstract

**Background:**

In limited resource settings, identification of factors that predict the occurrence of pulmonary hypertension(PH) in children with atrial septal defect(ASD) is important to decide which patients should be prioritized for defect closure to prevent complication. Echocardiography and cardiac catheterization are not widely available in such settings. No scoring system has been proposed to predict PH among children with ASD. We aimed to develop a PH prediction score using electrocardiography parameters for children with ASD in Indonesia.

**Methods:**

A cross-sectional study reviewing medical record including ECG record was conducted among all children with newly diagnosed isolated ASD admitted to Dr Sardjito Hospital in Yogyakarta, Indonesia during 2016–2018. Diagnosis of ASD and PH was confirmed through echocardiography and/or cardiac catheterization. Spiegelhalter Knill-Jones approach was used to develop PH prediction score. Accuracy of prediction score was performed using a receiver operating characteristic (ROC) curve.

**Results:**

Of 144 children, 50(34.7%) had PH. Predictors of pulmonary hypertension were QRS axis ≥120°, P wave ≥ 3 mm at lead II, R without S at V1, Q wave at V1, right bundle branch block (RBBB), R wave at V1, V2 or aVR > normal limit and S wave at V6 or lead I > normal limit. ROC curve from prediction scores yielded an area under the curve (AUC) 0.908(95% CI 0.85–0.96). Using the cut-off value 3.5, this PH prediction score had sensitivity of 76%(61.8–86.9), specificity 96.8%(91.0-99.3), positive predictive value 92.7%(80.5–97.5), negative predictive value 88.4%(82.2–92.6), and positive likelihood ratio 23.8(7.7–73.3).

**Conclusions:**

A presence of PH in children with ASD can be predicted by the simple electrocardiographic score including QRS axis ≥120°, P wave ≥3 mm at lead II, R without S at V1, Q wave at V1, RBBB, R wave at V1, V2 or aVR > normal limit and S wave at V6 or lead I > normal limit. A total score ≥ 3.5 shows a moderate sensitivity and high specificity to predict PH among children with ASD.

**Supplementary Information:**

The online version contains supplementary material available at 10.1186/s12887-023-04102-1.

## Background

Atrial septal defect (ASD) is one of the most common left to right shunt congenital heart diseases in children [1, 2]. ASD associated with pulmonary arterial hypertension (PH) presents with various clinical scenarios from large defect without or only mild PH, large defect with irreversible PH and a few case with small defect with severe disproportionate PH. Although ASD rarely cause PH in younger age, closure of ASD with PH in children still carries a significant morbidity and mortality despite after a proper assessment of operability of ASD in a low-to-middle-income countries [3]. Screening of PH in ASD is still challenging since the shunt is in diastolic phase and left to right shunt will remain even after elevated pulmonary arterial pressure thus cyanosis will not be detected clinically.

Electrocardiography (ECG) is a cheap and non-invasive method to predict structural abnormalities in the heart and widely available in almost all healthcare facilities including primary health care in low- and middle-income countries including Indonesia. Despite several discrepancies of using ECG for screening tools in patients with ASD and PH, development of scoring system for risk stratification in this clinical scenario is vital for accurate allocations of treatments in Indonesia with a very large archipelago and huge populations, but with very limited infrastructure, human, and financial resources. In order to make a risk stratification, we aimed to develop an accurate scoring system using ECG parameters to predict PH among children with ASD in Indonesia as similar to other low- and middle income-countries, which are not all patients have access to early diagnosis of congenital heart disease with PH confirmed by echocardiography and/or cardiac catheterization, which is only available in tertiary and referral hospitals.

In Indonesia, patients with ASD without PH are less priority for treatment, and it will be necessary to check for the appearance of PH during follow-up in facilities where echo is not readily available. Establishing the ECG scoring system still pose a challenge in converting numeric variable to points. However, by building this model, we do hope we can screen the ASD with or without PH and arrange for clinical treatment allocations including referral systems and decide which patients should be prioritized for defect closure to prevent further complication. As far as we are aware, this is among the first studies in children to evaluate the value of ECG in predicting PH in ASD. Further, no scoring system has been published to predict PH among children with ASD.

## Methods

A cross sectional study reviewing medical record including ECG record was conducted at the Department of Child Health at the Dr Sardjito Hospital in Yogyakarta, Indonesia. Patients aged < 18 years old with newly diagnosed isolated ASD attending the pediatric cardiology clinic and the general paediatric wards between 1st February 2016 to 31st December 2018, and echocardiography-confirmed ASD were included in the study. Patients were referred to our hospital mainly because of suspected CHD with or without heart failure. We excluded children with ASD who had other severe systemic problems, i.e. malignancy, chronic kidney disease, or autoimmune diseases, such as systemic lupus erythematosus and rheumatic heart disease.

Atrial septal defect was considered as small (> 3 mm to < 6 mm), moderate (≥ 6 mm to < 12 mm), or large (≥ 12 mm) defects [4]. Presence of genetic syndrome was defined when patients were diagnosed as having any congenital dysmorphic syndrome. The nutritional status was determined using criteria which derived from the standard of World Health Organization (WHO). Moderate malnutrition or wasting was defined as child’s weight was too low for their height, which was a Z score less than the two standard deviations below the median for weight for height adjusted for gender. Severe malnutrition or severely wasting was defined as weight for height below three standard deviations below the median for weight adjusted for gender.

Diagnostic criteria of PH were confirmed using transthoracic echocardiography (TTE) and/or cardiac catheterization including: a peak tricuspid regurgitation velocity > 3.4 m/s in the absence of pulmonary outflow obstruction or peak tricuspid regurgitation velocity of 2.9–3.4 m/s with presence of other echocardiographic PH signs and/or some degree of right-to-left shunt, all by TTE or mean pulmonary artery pressure (mPAP) > 20 mmHg and pulmonary vascular resistance index (PVRi) ≥ 3 WU [5, 6].

### Electrocardiography criteria

All children were performed 12 leads ECG at the day of CHD diagnosis was confirmed. The ECG examination used in this study was taken on the same day as the diagnosis of ASD and PH by using transthoracic echocardiography (TTE) and/or cardiac catheterization. If the subjects had more than one ECG, we used the ECG record that was taken on the same day as the diagnosis of PH using echocardiography or cardiac catheterization performed.

The ECG criteria that were used to predict PH including: (1) QRS axis: QRS axis ≥ 120°, (2) P wave at lead II of ≥ 3 mm, (3) R without S at V1 or S wave at V1 = 0, (4) Q wave at V1, (5) R at V1 and V2 > normal upper limit, and (6) right bundle branch block (RBBB). The latter criteria included: right axis deviation (RAD) with at least for the terminal portion of the QRS complex; the QRS duration **≥** 0.10 mm; and terminal slurring of the QRS complex that is directed to the right and usually, but not always, anteriorly wide and slurred S waves in leads I, V5, and V6 or terminal, slurred R′ in aVR and the right precordial leads (V4R, V1, and V2). The ECG cut off values were determined using the available cut off considering the age of patient [7].

### Statistics and Scoring System

The ECG parameters and outcome were compared using a chi-square test or Fisher’s exact test for categorical variables and Student’s t or Mann-Whitney U test for continuous variables. Continuous variables were transferred into a categorical variable based on the patients’ age cut-off point [7].

A prediction score was build based on natural logarithms of likelihood ratios (LR) of significant predictors. The first step was to calculate LR on each predictor. Predictors with LR + > 2 or LR- <0.5 were introduced in the logistic regression models following the methodology of Spiegelhalter Knill-Jones, allowing for likelihood ratios as results instead of odds ratios [8]. The second step was to take natural logarithms to calculate crude weight. This was followed by calculating adjusted weight based on crude weight and logistic coefficients. The adjusted weight was then used to make a prediction score.

The accuracy of the score was measured by the area under the curve (AUC). The optimum cut off point was determined by the best trade-off between false positives and false negatives from a receiver operating characteristic (ROC) curve. The internal validation of score was measured in 50 patients who were randomly assigned. Data analysis was performed using SPSS for Windows, Version 23.0 (SPSS Inc, Chicago, IL).

### Ethics

This study was conducted in accordance with the Declaration of Helsinki. The Medical and Health Research Ethics Committee, of the Faculty of Medicine, Public Health and Nursing, Universitas Gadjah Mada, Yogyakarta, Indonesia approved this study (KE/FK/1100/EC/2019). Because this study used secondary data, waiver of written informed consent was approved by the Medical and Health Research Ethics Committee, of the Faculty of Medicine, Public Health and Nursing, Universitas Gadjah Mada, Yogyakarta, Indonesia.

## Results

A total of 144 children with ASD were included, of those 50 (34.7%) children had developed PH. Of those with PH, only 22 (44%) children were performed cardiac catheterization. The clinical characteristics of the 144 children with ASD are shown in Table 1.

The types of syndromes were Down syndrome in 12 children, congenital Rubella syndrome in 2 children, and Noonan syndrome in 1 child. All children with Down syndrome (n = 12) were in the group of non-PH and none (n = 0) in the group of PH (p < 0.001).

**Table 1:** Baseline characteristic of 144 children with atrial septal defect

Parameters	Pulmonary hypertension (n = 50)	Non-pulmonary hypertension (n = 94)	p value
Age in months, median (min-max)	110.7 (2.1-212.6)	13.1 (2.1-211.5)	< 0.001
Age category, n (%)			
< 3 years-old	14 (28)	59 (62.8)	< 0.001
≥ 3 years-old	36 (72)	35 (37.2)	
Sex, n (%)			
Female	31 (62)	60 (63.8)	0.857
Male	19 (38)	34 (36.2)	
Diameter of ASD in mm, mean (±SD)	19.3 (±10.5)	9.4 (±7.2)	< 0.001
Size of ASD, n (%)			
Small	7 (14)	44 (46.8)	< 0.001
Moderate	8 (16)	33 (35.1)	
Large	35 (70)	17 (18.1)	
Type of ASD, n (%)			
Secundum	46 (92)	94 (100)	
Primum	2 (4)	0 (0)	0.021
Sinus Venosus	2 (4)	0 (0)	
Nutritional status, n (%)			
Normal	32 (64)	66 (70.2)	0.020
Moderate malnutrition	9 (18)	24 (25.5)	
Severe malnutrition	9 (18)	9 (4.3)	
Syndrome, n (%)			
Yes No	4 (8) 46 (92)	12 (12.8) 82 (87.2)	0.579

ASD = atrial septal defect; SD = standard deviation

We established a predictive scoring system using the Spiegelhalter-Knill-Jones method. Table 2 shows QRS axis ≥ 120°, P wave ≥ 3 mm at lead II, R without S at V1, Q wave at V1, RBBB, R wave at V1, V2 or aVR > normal limit and S wave at V6 or lead I > normal limit of age were associated with PH in children with ASD, which were introduced in logistic regression models. Table 3 shows the adjusted weights for QRS axis ≥ 120°, P wave ≥ 3 mm at lead II, R without S at V1, Q wave at V1, RBBB, R wave at V1, V2 or aVR > normal limit and S wave at V6 or lead I > normal limit to diagnose PH by logistic determination.

**Table 2:** Diagnostic value and likelihood ratio for electrocardiographic parameters to predict pulmonary hypertension in ASD

Parameters	Pulmonary hypertension n = 50	Non-Pulmonary hypertension n = 94	Sensitivity (%)	Specificity (%)	PPV (%)	NPV (%)	p value
QRS axis			52.0	79.8	57.8	75.8	0.225
≥ 120°	26	19					
< 120°	24	75					
P wave at lead II			50.0	96.8	89.3	78.4	0.003
≥ 3 mm	25	3					
< 3 mm	25	91					
R without S at V1			56.0	93.6	82.4	80.0	0.019
Yes	28	6					
No	22	88					
Q wave at V1			36.0	97.9	90.0	74.2	0.042
Yes	18	2					
No	32	92					
RBBB			62.0	90.4	77.5	81.7	0.027
Yes	31	9					
No	29	85					
R at V1, V2 or aVR			62.0	79.8	62.0	79.8	0.044
High	31	19					
Normal	19	75					
S at V6 or Lead I			74.0	74.5	60.7	84.3	0.560
High	37	24					
Normal	13	70					
R/S ratio at V1 or V2 > N, or V6 < 1			86.0	43.6	44.8	85.4	0.398
Abnormal	43	53					
Normal	7	41					

PPV = positive predictive value; NPV = negative predictive value; RBBB = right bundle branch block

**Table 3:** Adjusted weights for prediction of pulmonary hypertension by logistic determination

Parameters		Pulmonary hypertension (n = 50)	Non-Pulmonary hypertension (n = 94)	Likelihood ratio		Crude weight (Logistic coefficient)	LR*log coefficient	Adjusted weight (log natural)	
QRS axis	≥ 120°	26	19	LR+	2.57	2.1	5.4	2	
	< 120°	24	75	LR-	0.60	0.5	0	0	
P wave at lead II	≥ 3 mm	25	3	LR+	15.67	11	172	5	
	< 3 mm	25	91	LR-	0.52	0.1	0	0	
R without S at V1	Yes	28	6	LR+	8.77	6	53	4	
	No	22	88	LR-	0.47	0.2	0.1	-2	
Q wave at V1	Yes	18	2	LR+	16.92	8	135	5	
	No	32	92	LR-	0.65	0.1	0	0	
RBBB	Yes	31	9	LR+	6.48	4.7	30	3	
	No	19	85	LR-	0.42	0.2	0.1	-2	
R wave at V1, V2 or aVR	High	31	19	LR+	3.07	3	9.2	2	
	Normal	19	75	LR-	0.48	0.3	0.1	-2	
S at V6 or lead I	High	37	24	LR+	2.90	1.5	4.4	1	
	Normal	13	70	LR-	0.35	0.7	0.2	-1	
R/S ratio at V1 or V2 > N or V6 < 1	Abnormal	43	53	LR+	1.53	0.5	0	0	
	Normal	7	41	LR-	0.32	2	0.6	0	

RBBB = right bundle branch block; LR = likelihood ratio; N = normal

**Fig. 1:**
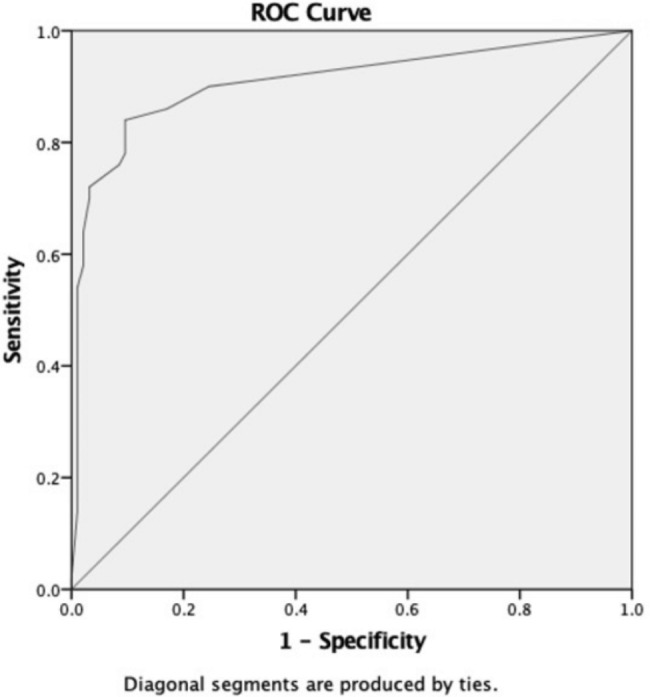
ROC curve on diagnostic score for pulmonary hypertension

The maximum score was 22 and the minimum was − 7. Figure 1 shows the ROC curve of this prediction score had an AUC of 0.908 (95% CI: 0.85–0.96). The cut off value of 3.5 had optimal sensitivity and specificity in diagnosing PH. This cut off value of 3.5 showed sensitivity 76.0% (61.8–86.9), specificity 96.8% (91.0-99.3), positive predictive value (PPV) 92.7% (80.5.2–97.5), negative predictive value (NPV) 88.4% (82.2–92.6), LR + 23.8 (7.7–73.3), and LR- 0.25 (0.15–0.41) with the accuracy of 89.6% (83.4–94.1).

The internal validation was done to validate this prediction score with the AUC of validated score 0.930 (0.859-1.000). Using the cut value of 3.5, the sensitivity was 80.0% (59.3–93.2), specificity 92.0% (74.0–99.0), PPV 90.9% (72.3–97.5), NPV 82.1% (67.6–91.0), LR + 10.0 (2.6–38.3), LR- 0.22 (0.10–0.48) with accuracy 86.0% (73.3–94.2).

## Discussion

This study explored the performance of ECG in predicting PH among 144 children with ASD at a tertiary referral hospital in Yogyakarta, Indonesia. We have demonstrated the high prevalence of PH (34.7%) in children with ASD, which is reasonable because of the high proportion of delayed diagnosis of CHD in our setting [1]. Further, the results of this study indicate that ECG parameters can be used to predict the presence of PH by using QRS axis ≥ 120°, high P wave at lead II, R without S at V1, Q wave at V1, RBBB, R wave at V1, V2 or aVR > normal limit and S wave at V6 or lead I > normal limit. A total score ≥ 3.5 shows a moderate sensitivity and high specificity to diagnose PH among children with ASD.

In Indonesia, screening of CHD in children has not been routinely performed despite a considerable burden of CHD exists [1]. A study in Yogyakarta, Indonesia found that a significant delayed diagnosis of CHD in 6 out of 10 children, most with severe complications including PH in 15.8% patients [1]. Another study in Yogyakarta did a screening of CHD among 6116 first grade elementary students using cardiac auscultation and 12-lead electrocardiogram. The screening program was feasible and CHD was found in 0.29% [9]. Another study in the same city performed a screening of critical CHD using pulse oximetry among 1452 neonates and critical CHD is seen in 6 of 1000 neonates [10]. Routine cardiac catheterization did not recommend before ASD closure in children, due to its cost and non-applicability in limited resources setting. In resource limited settings such as in Indonesia, and the treatment priority for ASD patients is high for those cases that are complicated by PH, and the absence of PH means that they have to wait for treatment, then ASD patients without PH have a low priority for treatment and will be followed up at a medical facility near their place of residence. During the follow-up, echocardiography is not possible frequently due to resource issues and access to hospitals that can perform echocardiography, but ECG can be performed at a medical facility near where the patient lives, and therefore, it is very useful to be able to use our scoring system to explore the possibility of the appearance of PH.

Although ECG is more sensitive in younger patients, an assessment of the ECG as a screening test for large atrial septal defects in children has been proved as unreliable for screening of ASD in a study. Moreover, the sensitivity of ECG in detecting right ventricular hypertrophy in ASD has been reported to vary from 60 to 90% in selected populations. However, among patients with ASD, ECG can be used to estimate whether they have developed PH. Some studies had been conducted to evaluate the performance of ECG in predicting PH in atrial septal defect, but mostly in adult population [11–13].

An ECG is still widely used in clinical setting as a simple, efficient and inexpensive method as part of screening tools of congenital heart disease. In general, common ECG patterns seen in PH include right atrial abnormalities, right axis deviation and right ventricular hypertrophy with strain pattern [14]. An ECG had low specificity in the diagnosis of ASD because ECG of patients with ASD can be normal. But ECG is more accurate than chest X-ray in the detection of cardiac chamber enlargement and heart diseases in general [15].

The hemodynamic of isolated ASD depends on the direction of the shunt, which is left to right shunt at the atrial level. This shunt created volume overload and cardiac chambers dilatation, specifically right atrium (RA), right ventricle (RV), and main pulmonary artery. The most accepted ECG criteria of RA enlargement (RAE) is a high P wave at lead II, III, and aVF. In this study, RAE alone (P wave ≥ 3 mm at lead II) on ECG had low sensitivity of 50.0%, but high specificity of 96.8% for diagnosing PH in children with ASD. Previous studies also showed that the P wave > 2.5 mm at lead II was very specific (100.0% specificity), but the sensitivity was very low to detect RAE [16, 17]. More specifically, P amplitude at lead II increases as a result of progressive RV hypertrophy-associated diastolic dysfunction, and RV dilatation-associated tricuspid regurgitation in PH patients [18]. In contrast, in patient with asymptomatic ASD, RAE was found in 6% patients [19].

Another ECG parameter to predict PH is the presence of abnormal Q wave (qR wave) at V1. RV disfunction and dilation with diastolic interventricular septum flattening due to the increase of pulmonary vascular resistance are reflected by qR wave in V1. qR pattern at V1 was present in almost half of PH and correlated with worse exercise capacity and higher N-terminal pro b-type natriuretic peptide (NT-proBNP) [20]. Our study also reported the presence of qR wave at V1 alone was poorly sensitive in ASD with PH compared to ASD without PH (36.0% vs. 2.1%). However, its ECG pattern had the highest specificity (97.9%) compared to other parameters for PH in ASD. A previous study showed the presence of Q wave at V1 was a sign of advanced PH and significant poor prognostic factor. Further, abnormal Q wave at V1 also had highest positive predictive value for diagnosing PH of 90.0% [21].

In our population, half patients had QRS axis ≥ 120, with specificity is 79.8%, PPV is 57.8% and NPV is 75.8%. In contrast, a previous study conducted among young children with ASD reported QRS axis ≥ 120° (right axis deviation/RAD) was associated with high PPV and modest NPV [22]. Sawada et al. reported that RAD was found in 79% patient with PH [23]. An RAD is a reflection of RV hypertrophy due to volume (e.g. in ASD) or pressure (e.g. in PH or pulmonary stenosis) overload. Therefore, in case of differentiating ASD with or without PH, RBBB pattern should be explored. In our study, RBBB was found in patients with PH compared to those without PH (62.0% vs. 31.0%). The sensitivity and specificity of RBBB on ECG for diagnosing PH in children with ASD were 62% and 90.4%, respectively. A previous study showed that RBBB is correlated with RV overload (high Qp/Qs ratio) in children with ASD [24].

An RAE and volume type of RV hypertrophy itself are indicators for ASD and the presence of these might be considered for patients to be referred for the diagnosis of ASD. However, we also would like to assess whether these parameters also predicted the occurrence of PH. In volume type of RV hypertrophy, we may consider that the patient having volume overload of ASD, but in this study we also demonstrate that these parameters also predicted signs for PH. Therefore, the use of this ECG scoring system may also provide an overview of the possible presence of PH in ASD patients.

The sensitivity and specificity of the presence of high amplitude R wave at V1, V2 or aVR was 62.0% and 79.8%, respectively. High S wave at V6 or lead 1 and R/S ratio at V1,V2 or V6 had higher sensitivity (74.0% and 86.0%), but lower specificity (74.5% and 43.6%) compared to other parameters. In contrast, the detection of RV hypertrophy on the ECG is highly specific, but has too low of a sensitivity to exclude PH in general adult population [25]. This difference might be due to the different population from our study. The presence of R without S wave at V1 has good positive and negative predictive value, 82.4% and 80.0% respectively. Abnormal R/S ratio at V1, V2 or V6 had highest sensitivity (86.0%) and NPV (85.4%) to diagnose PH. Another study in children population showed ECG has limited value as a screening tool for RVH because of its relatively low sensitivity and PPV with the sensitivity 69.0% and PPV 67.0% [26].

On the basis of individual parameter of ECG, we found marked variations of sensitivity and specificity to diagnose PH. Therefore, we combined each of the parameters and found that a total score ≥ 3.5 could predict PH in children with ASD with sensitivity 76.0%, specificity 96.8%, PPV 92.7%, NPV 88.4%, LR + 23.8, and LR- 0.25. This study is among the first study to evaluate an ECG-based score for prediction of PH in children with ASD. We showed that a higher score is associated with a higher incidence of PH and that an ECG score ≥ 3.5 has good discriminatory power to predict PH in ASD patient in the ROC analysis. The ROC analysis shows high sensitivity, specificity and accuracy to predict PH. While the advantages of the ECG score are simple, efficient and cost effectiveness; our ECG-based score had both high sensitivity and specificity (76.0% and 96.8%) for diagnose PH, probably due to high prevalence of PH in our study population.

This study was limited to a tertiary referral hospital in Yogyakarta, Indonesia and whether these results can be generalized needs to be evaluated in the future, since the study was conducted in a tertiary hospital considering the difference of high prevalence (pre-test probability) of PH in our setting and we did not include external validation cohort. However, we consider that this study shines the light of the use of ECG to predict PH in children with ASD. Another limitation is that not all the cases where PH was confirmed by the cardiac catheterization, a gold standard. The age and size as well as Qp/Qs may contribute to the difference of ECG findings and can be as predictors of PH, however no Qp/Qs data by echocardiography were available. The low rate of performing cardiac catheterization among children with ASD in this study because this procedure was only conducted when the defect was planned to close using transcatheter ASD closure or by surgical procedure. Not all children with PH in ASD were performed closure of the defect in our setting. This is because we only performed transcatheter closure in children with ASD at around 5 years of age or the body weight of 15 kg. While surgical closure of ASD in our setting is only done in patients with at least 6 kg of body weight. Other than that, we refer the patients to national cardiac centres in our capital city of Jakarta to close the ASD surgically. Another reason for not performing surgery is that a social factor related to parents or caregivers of rejecting to perform cardiac surgery when the transcatheter closure is not suitable. Another limitation includes we did not rule out other types of PH including of pulmonary vascular obstructive diseases, lung disease, collagen disease or other causes. We consider the cause of PH in our study was associated with the presence of ASD. Based on our unpublished study in 2016 to 2018 in the same setting, approximately 95% children with PH associated with CHD. Further, in this current study we showed that all children with Down syndrome were in the group of non-PH as children with Down syndrome may have increased risk of PH due to multiple factors not only CHD.

In conclusion, the presence of PH in children with ASD can be predicted with ECG parameters including QRS axis ≥ 120°, P wave ≥ 3 mm at lead II, R without S at V1, Q wave at V1, RBBB, R wave at V1, V2 or aVR > normal limit and S wave at V6 or lead I > normal limit. The presence of PH in children with ASD can be predicted by these simple ECG parameters especially in limited resource settings. A total score ≥ 3.5 shows a moderate sensitivity and high specificity to diagnose PH among children with ASD. This ECG scoring system will save more time and resources in treating children with ASD in limited resources health care facilities.

## Electronic supplementary material

Below is the link to the electronic supplementary material.

Supplementary Material 1

## Data Availability

All data generated or analysed during this study are included in this published article [and its supplementary information files].

## List of abbreviations

ASD: Atrial Septal Defect
AUC: Area Under the Curve
CHD: Congenital Heart Disease
ECG: Electrocardiography
LR: Likelihood Ratio
mPAP: Mean pulmonary artery pressure
NPV: Negative Predictive Value
NT-Pro BNP: N-terminal pro b-type natriuretic peptide
PH: Pulmonary hypertension
PPV: Positive Predictive Value
PVRi: Pulmonary vascular resistance index
RA: Right Atrium
RAD: Right Axis Deviation
RAE: Right Atrium Enlargement
ROC: Receiver Operating Characteristic
RBBB: Right Bundle Branch Block
RV: Right Ventricle
RVE: Right Ventricular Hypertrophy
Sn: Sensitivity
Sp: Spesificity
TTE: Trans-thoracic Echocardiography
WHO: World Health Organization
